# Medical Jousting in Research Publications: A Call for Ethical Discourse

**DOI:** 10.4274/TJAR.2025.251893

**Published:** 2025-12-22

**Authors:** Ashwin Mani, Sukriti Jha, Prakash Gyandev Gondode, Sakshi Duggal

**Affiliations:** 1 All India Institute of Medical Sciences, Department of Anaesthesiology, Pain Medicine and Critical Care, New Delhi, India

**Keywords:** Ethics, letter to editor, medical jousting, publication ethics, scholarly criticism

Dear Editor,

The “Letter to the Editor” (LOE) section in academic journals has a rich history dating back to the 15^th^ century when van Leeuwenhoek^1^ wrote regularly to the Royal Society of London, sharing his landmark scientific observations. Over time, this practice evolved into a formalised forum for intellectual discourse. It became a platform to critique published articles, offer fresh insights, and draw attention to overlooked aspects of studies.^2, 3^ This section has played a pivotal role in advancing scientific dialogue, fostering transparency, encouraging collaboration, and driving the refinement of knowledge. Functioning as a form of post-publication peer review, it ensured authors were held accountable for their work. However, despite its intent to promote constructive debate, this platform has at times been misused, with instances of what can be described as “medical jousting.”

Medical jousting, traditionally observed in clinical practice as verbal criticism or disputes among healthcare professionals, is increasingly evident in research publications. In this context, it manifests as authors leveraging letters to the editor or commentary within articles to discredit or undermine previous research. This practice deviates from the original purpose of scholarly critique, which is to enhance understanding, not to disparage.

In academic journals, medical jousting can take several forms. Critiques may lack balance, omit opposing research, or use dismissive language. Authors sometimes misrepresent studies or employ hostile, unprofessional responses, including personal attacks, instead of constructive discourse, undermining scholarly communication and the advancement of knowledge in the field.

Such practices have far-reaching implications. They not only erode trust in academic publishing but also discourage researchers—especially early-career scientists—from participating in scientific discourse for fear of undue criticism. Furthermore, they create a toxic environment that stifles collaboration, which is the cornerstone of medical progress.^4^

The style of writing in these letters is often informal, with highly subjective language that appears judgmental. Magnet and Carnet^5^ observed that the lexicon used frequently leaned towards the disparaging, occasionally even crossing into the derogatory. They noted the presence of emotionally charged verbs, adjectives, and negative prefixes such as *mis-* and *dis-*. Some authors even employed sarcasm and banter in their tone. At least 56% of the original authors chose not to respond to these letters, possibly due to their personal and critical nature. Interestingly, a survey conducted by the researchers revealed that readers often found LOE concise and manageable for regular reading. However, many admitted they did not always follow up by reading the original article. This raises a significant concern: a harshly worded letter could potentially create a bias among readers toward the original author, influencing perceptions unfairly.^5^

Addressing medical jousting in research publications requires a concerted effort from authors, reviewers, and journal editors. The original purpose of letters to the editor—to act as a bridge between published research and further inquiry—remains as important as ever. However, for it to fulfil this role, it must be used responsibly.

## References

[1] van Leeuwenhoek A (1679). A letter of Mr. Leeuwenhoek to Dr. G., containing an account of his observations lately made of vast numbers of animals in Semine Animalium.. Philos Collect R Soc Lond.

[2] Divatia JV (2018). Comments on published article: a valuable resource.. Indian J Anaesth.

[3] Goneppanavar U, Karippacheril JG, Magazine R (2016). Critical appraisal of published literature.. Indian J Anaesth.

[4] Dkhar SA (2018). Letter to editor: its importance and drawbacks.. Int J Community Med Public Health.

[5] Magnet N, Carnet D (2006). Letters to the editor: still vigorous after all these years? A presentation of the discursive and linguistic features of the genre.. Engl Specif Purp.

